# Influence of time since naturalisation on socioeconomic status and low birth weight among immigrants in Belgium. A population-based study

**DOI:** 10.1371/journal.pone.0220856

**Published:** 2019-08-15

**Authors:** M. Sow, C. Schoenborn, M. De Spiegelaere, J. Racape

**Affiliations:** 1 Université Libre de Bruxelles (ULB), Research centre in Health Policies and Health Systems, School of Public Health, Brussels, Belgium; 2 Université de Montréal, School of Public Health, Montréal, Québec, Canada; 3 Université Libre de Bruxelles (ULB), Research centre in Epidemiology, Biostatistics and Clinical research, School of Public Health, Brussels, Belgium; 4 Chair in Health and Precarity, Université Libre de Bruxelles (ULB), Médecins du monde, Brussels, Belgium; Erasmus Medical Center, NETHERLANDS

## Abstract

**Background:**

Increasingly studies show that immigrants have different perinatal health outcomes compared to native-born women. Nevertheless, we lack a detailed examination of the combined effects of maternal immigrant trajectory and socioeconomic status on perinatal outcomes. Our objective was to analyze the influence of time since naturalization on low birth weight and maternal socioeconomic status in Belgium.

**Methods:**

The data came from the linkage between the Brussels birth and death registers, the national register of migrant trajectories and the social security register for the years 2004–2010. We used logistic regression to estimate the odds ratios of the associations between low birth weight (LBW) and time since naturalization, by nationality groups, taking into account socioeconomic status (SES), parity and maternal age.

**Results:**

Data relate to all singleton births to Belgian, Maghrebi, Sub-Saharan African and Turkish women (n = 76 312). The results show an U-shaped of LBW according to time since naturalization for all migrant groups. LBW declines for women naturalized since less than one year and increases significantly thereafter (p<0.0001). In parallel, we observe an increase of SES among all migrant groups. Compared to Belgians, we found a lower risk of LBW among women from Maghreb (p<0.0001) and this protection is maintained even after 10 years since naturalization. In contrast, the risk of LBW for Sub-Saharan African and Turkish mothers is lower than for Belgians after one year of naturalization but similar to that of Belgians after 10 years of naturalization.

**Conclusion:**

Our results show that, despite an improvement of their SES, LBW increases among Maghrebi, Sub-Saharan African and Turkish women with time since naturalization. Mothers from Maghreb have lower rates of LBW compared to Belgians and maintain their protection even after more than 10 years of having acquired the Belgian nationality. Additional studies need to be carried out in order to gain a better understanding of the association between migration trajectories, SES and perinatal health of immigrants.

## Introduction

Among perinatal health indicators, low birth weight (LBW) is one of the most often used to estimate the impact of a combination of different circumstances on newborns’ health. LBW influences health in the neonatal period, in childhood and also in adulthood [1–3]. It is the consequence of premature birth and/or intrauterine growth restriction [2]. Many factors, acting at different levels, influence birth weight: physiology, maternal health behaviors, social determinants and access to health care [4,5]. Increasing studies show that immigrants have different perinatal health outcomes compared to native women. The magnitude and direction of these differences in adverse pregnancy outcomes vary in different contexts according to ethnicity or immigrants region of origin [6–9]

The “epidemiological paradox”, also known as the Mexican paradox describes the fact that despite low socioeconomic status, immigrants have better perinatal outcomes than the native population [10–12]. This observation has been revised by several studies which show that the paradox depends on the perinatal outcomes and the migrant groups that are being studied [8,13–17]. For example, in Belgium, we observe lower rates of LBW among women from Maghreb and Turkey, but higher rates among sub-Saharan Africans compared to Belgian mothers even after adjusting for their SES [14,15,18].

Furthermore, some studies show that migrants’ perinatal health varies with the time spent in the host country. Among migrants, perinatal health outcomes such as LBW and preterm birth, deteriorate with the time spent in the host country [7,8,19,20]. Similar observations have been made for other health outcomes such as overweight/obesity [21], diabetes, and breast cancer [22]. It has been hypothesized that migrant health tends to converge over time with the pattern observed in the native-born population [23]. The deterioration of migrants’ health with duration of residence is somewhat paradoxical given that there is often an improvement in their socio-economic level. These studies show the importance of taking into account the time spent in the host country when analysing the risk of adverse birth outcomes among immigrants.

In Belgium, previous studies analyse the influence of SES and migration on perinatal outcomes [14,18,24]. The results have shown that perinatal mortality varies according to maternal nationality but these differences do not persist after adopting the Belgian nationality [14]. Our results have shown a protective effect of migration in relation to perinatal mortality and LBW among women of low SES but not among women with higher SES [18]. We do not observe the classic social gradient according to maternal education in perinatal outcomes among migrant groups [24]. These studies underline the importance of taking into account socioeconomic status in order to understand more fully the relationship between migration and perinatal outcomes.

With a new linked database, to our knowledge the first one in Belgium, it was possible to analyse additional socio-economic and migration variables such as the time since naturalization. The aim of this study was to analyze the influence of migrant’s time since naturalization on their SES and on their risk of LBW. More specifically, is time since naturalization associated with an improvement of the mother's socioeconomic status and a reduction of the risk of LBW?

## Materials and methods

### Study population and data

The data we used came from the linkage between three administrative databases: the birth and death certificates taken from the Belgian civil registration system, the national register, and the social security register (BCSS Crossroads Bank for Social Security). The data relates to all singleton babies born alive between 1st January 2004 and 31st December 2010, whose mothers were living in Brussels. In Belgium, all live births and foetal deaths from 22 completed weeks of gestation or a birth weight >500 grams must be registered. The data assessment and quality of birth and death certificates are managed by two perinatal epidemiology centers, in collaboration with maternity wards and civil registration services [18,25]. The BCSS database electronically gathers the socioeconomic data originating from social security administrations in Belgium. The national registry is a centralized file that contains the identification data of the Belgian citizens residing in Belgium or abroad and of any other individuals who legally reside in Belgium, as well as some information on the people who have applied for refugee status.

The linkage between the 3 databases has been carried out by the Directorate-General Statistics and Economic Information and by the Crossroads Bank for Social Security, after obtaining the approval of the Privacy Protection Commission. The data collection was carried out by Statistics Belgium and was exempted by law from requiring ethical approval. The process of obtaining linked databases from the Belgian civil registration system is regulated by the Belgian Commission for the Protection of Privacy. After approval of this Commission, Statistics Belgium provided the data for the period 01/01/2004 to 31/12/2010.

### Definitions of the exposures and outcomes

#### Outcome variables

We considered a birth weight to be low (LBW) when below 2,500g and prematurity a birth before 37 weeks of gestation. LBW is an indicator that has the advantage of being easily comparable between different contexts (easy to measure, less measurement error). LBW also appears more ‘sensitive’ to certain socio-economic conditions. For example, it is more often associated with low income than preterm or small for gestational age [26]. LBW was analysed according to the mother’s migration trajectory (maternal nationality at birth and at delivery, adoption of the Belgian nationality and time since adopting the Belgian nationality), socioeconomic indicators (maternal education, household income) and single parenthood. Maternal age and parity were used as adjustment variables.

#### Maternal nationality

Maternal nationality is recorded as declared at the civil registration service. We defined maternal nationality of origin as her nationality at her own birth, and the current nationality as the nationality at her child’s birth. We classified nationalities into the six most represented groups in the country, namely Belgium, European Union (EU), Turkey, Maghreb, and sub-Saharan Africa. We did not study mothers from the EU because too few of them have been naturalized. The proposed categorisation takes into account the mapping of the various parts of the world, the distribution of births in Brussels and the results of previous studies on perinatal health in Brussels [14,15]. In Belgium, North African immigration comes mainly from the Maghreb.

#### Time since naturalisation

Among mothers who were not Belgian when they were born, we defined naturalisation as the mother’s adoption of the Belgian nationality before giving birth.

In Belgium, the nationality can be acquired in two ways: by statement or naturalization and are open to adults who meet a series of requirements related to place of birth, attachments to Belgium and legal residence (for at least three years or two (for refugees) until 2010). Most commonly, immigrants acquire the Belgian nationality through statement (97%) and the first condition to make this declaration is the length of residence [27]. Before 2012, the requirement to start the procedure was to have resided in Belgium for 7 years. The procedure does not depend on cultural or language knowledge, nor on medical conditions. (“https://diplomatie.belgium.be/fr/Services/services_a_letranger/nationalite,” n.d.). Time since naturalization is an important variable to understand perinatal health among immigrants in Belgium. In our previous works we have shown that the acquisition of the Belgian nationality decreases adverse pregnanacy outcomes among migrant groups in Belgium [14,18]. Studying the time since naturalisation, allows us to study the adoption of the Belgian nationality in more detail, by diving it into short (less than 1 year), medium (between 1 and 10 years) and long term (more than 10 years).

Time since naturalisation was categorized into less than 1 year, between 1 to 5 years, between 5 to 10 years and more than 10 years after the adoption of the Belgian nationality. Finally, for mothers from Maghreb, sub-Saharan Africa and Turkey, we created 5 categories which considered the mother’s nationality of origin, the acquisition of the Belgian nationality and the length of this acquisition.

M**aternal education** was organised into five categories: superior (university or higher education), upper secondary (completed secondary school), lower secondary (up to the third completed year of secondary school), lower primary (completed primary or less), and other.

**Household income** includes earned incomes and replacement incomes collected yearly. In the database, we have the equivalent income for households, by deciles. These deciles are based on the distribution of incomes of all Belgian households, which we categorized into quintiles. The poorest quintile (Q1) is close to the risk-of-poverty threshold. Based on income quintiles, three categories of households were created: households at risk of poverty (Q1), median income households (Q2 and Q3), and high-income households (Q4 and 5).

**Household situation**, based on the situation declared to the administration, this variable distinguished 2 categories: children whose parents live together (married or not) and those who are born into a single-parent household.

### Statistical analysis

We analysed how the proportions of mothers with higher maternal education, household income in the first quintile, and single mothers varied by time since naturalisation by using p-trend Chi2. We used Pearson Chi2 to compare those proportions between Belgian mothers and immigrant mothers who have acquired the Belgian nationality for more than 10 years. We used logistic regression to estimate odds ratios (ORs) for the associations between maternal nationality and LBW and prematurity (not shown) according to time since naturalisation. First, the crude associations between LBW and naturalisation length were estimated. Thus, a series of multivariate models were developed to assess the effect of parity, maternal age, maternal education, household situation and household income as potential confounding variables.

The adequacy of the model was checked by the Hosmer and Lemeshow test. We present the odds ratios derived from the logistic regressions and the p-values for the Wald Chi^2^ test. The significance level was set at alpha = 0.05, and all the analyses were performed using Stata 14 software.

## Results

### Characteristics of mothers and new-borns, according to maternal nationality and length of naturalization

Data relate to all singleton births among Belgian, Maghrebi, Sub-Saharan African and Turkish women in Brussel from 01/01/2004 to 31/12/2010 (n = 76 312).

#### Socio-economic deprivation among immigrants

Immigrant mothers were significantly more socio-economically disadvantaged than Belgian mothers (whether naturalized or not) (Table 1). Belgian mothers are less likely to have completed only primary school (1,5% versus 24,8%, 19,4% and 32,3% for Maghreb, sub-Saharan and Turkish non-naturalized mothers respectively). They have the highest proportion of higher education diplomas (51,9% versus 7,4%, 13,4% and 4,8% for Maghreb, sub-Saharan and Turkish non-naturalized mothers respectively), and the lowest proportion of income in the first quintile (18,4% versus 64,8%, 69,5% and 60,3% for Maghreb, sub-Saharan and Turkish non-naturalized mother respectively) compared to immigrants. Sub-Saharan African mothers are more likely to have completed secondary school compared to mothers from Maghreb or Turkey (Table 1).

**Table 1:** Distribution of singleton births by maternal characteristics and time since naturalization.

N = 76 143	Belgium n = 27265	Maghreb (n = 10744)	Maghreb naturalized (years)				Sub-Saharan Africa (n = 4989)	Sub-Saharan Africa naturalized (years)				Turkey (n = 1839)	Turkey naturalized (years)			
			< 1 (n = 3754)	1 < 5 (n = 4718)	5 < 10 (n = 6197)	≥ 10 (n = 7295)		< 1 (n = 1377)	1 < 5 (n = 1708)	5 < 10 (n = 1163)	≥ 10 (n = 669)		< 1 (n = 536)	1 < 5 (n = 836)	5 < 10 (n = 1501)	≥ 10 (n = 1552)
**Parity (%)**																
**Nulliparity**	56.47	47.34	32.82	28.61	35.36	36.48	47.37	39.16	37.4	43.44	51.05	41.17	24.19	27.07	32.21	40.21
**≥ 3**	1.46	4.2	4.22	9.34	7.07	5.37	4.28	3.97	5.77	5.09	3.29	1.93	3.43	5.61	3.79	2.64
**Maternal age (%)**																
**<20**	2.64	3.34	1.76	1.0	1.55	1.04	3.49	1.67	2.22	4.56	3.44	7.61	4.66	2.39	3.86	1.61
**≥40**	3.9	4.12	5.38	7.33	4.86	3.32	2.51	3.85	5.44	9.03	6.58	1.25	1.12	1.91	2.07	1.29
**Maternal education (%)**																
**Primary**	1.53	24.75	15.95	14.66	9.42	5.82	19.38	10.16	7.47	6.68	2.88	32.28	29.21	19.13	14.3	9.13
**Secondary inferior**	14.17	24.15	18.04	23.5	23.46	27.51	25.38	17.11	19.64	24.83	24.7	24.17	22.1	22.02	21.72	28.35
**Secondary superior**	27.21	31.98	43.89	40.3	42.87	40.54	33.07	45.39	39.8	33.68	34.7	29.18	35.58	41.76	44.65	39.87
**Higher**	51.89	7.39	8.4	9.32	13.7	19.52	13.39	18.2	24.57	27.43	33.33	4.84	3.75	6.02	9.69	15.73
**Other**	5.2	11.73	13.73	12.22	10.54	6.61	8.78	9.14	8.52	7.38	4.39	9.53	9.36	11.07	9.63	6.93
**Single mother (%)**																
**yes**	15.63	8.17	8.9	10.31	12.94	16.52	38.87	38.9	40.02	38.34	31.69	6.88	6.43	12.62	15.25	15.9
**no**	79.92	86.66	87.71	87.51	83.71	79.67	50.58	56.34	55.09	53.36	57.69	67.71	72.21	74.03	71.86	73.69
**unknown**	4.46	5.16	3.39	2.17	3.35	3.81	10.55	4.76	4.88	8.3	10.62	25.41	21.36	13.35	12.89	10.41
Incomes* **(%)**																
**Q1**	18.36	64.81	59.61	54.41	45.38	38.4	69.54	56.06	54.97	50.05	40.92	60.31	53.78	53.46	47.48	42.88
**Q2-Q3**	27.92	33.26	37.0	40.32	45.66	46.1	26.46	34.39	34.8	36.71	36.13	36.84	44.32	43.63	46.02	43.65
**Q4-Q5**	53.72	1.93	3.39	5.27	8.96	15.51	4.00	9.55	10.22	13.25	22.95	2.85	1.89	2.92	6.5	13.48

*without 2004

#### Socio-economic deprivation decreases with time since naturalization

Among all immigrants, the proportion of women having completed higher education increases significantly with time since naturalization, from less than one year of naturalization to more than 10 years of naturalization (p<0,0001) (Table 2): 8,4% to 19,5% (Maghreb), 18,2% to 33,3% (sub-Saharan Africa) and 3,8 to 15,7% (Turkey). Even after 10 years of naturalization the proportions of immigrant women having a higher education diploma remain significantly lower than the proportion of Belgian mothers (51,9%), (p<0,0001). A similar pattern is observed for household income (Table 2): The proportion of households at risk of poverty decreases significantly with time since naturalisation, from less than one year of naturalization to more than 10 years of naturalization (p<0,0001): 59,6% to 38,4% (Maghreb), 56,1% to 40,9% (sub-Saharan Africa) and 53,8% to 42,9% (Turkey). These proportions remain significantly higher compared to Belgian mothers (18,4%) even after 10 years of naturalization (p<0,0001).

**Table 2:** Higher maternal education, poverty line (Q1 income) and single mother according to time since naturalization.

			Naturalization length (years)									
			<1 year		1<5 years		5<10 years		≥ 10 years		ptrend	Belgium vs ≥ 10 years of naturalization
	n	%	n	%	n	%	n	%	n	%		
**Higher maternal education**												
**Belgium**	14 082	51.9										
**Maghreb**			314	8.40	436	9.32	841	13.70	1414	19.52	<0.0001	<0.0001
**Sub-Saharan Africa**			249	18.20	418	24.57	316	27.43	220	33.33	<0.0001	<0.0001
**Turkey**			20	3.75	50	6.02	145	9.69	243	15.73	<0.0001	<0.0001
**Income Q1**												
**Belgium**	4 168	18.4										
**Maghreb**			1616	59.61	2065	54.41	2389	45.38	2488	38.40	<0.0001	<0.0001
**Sub-Saharan Africa**			546	56.06	785	54.97	529	50.05	239	40.92	<0.0001	<0.0001
**Turkey**			199	53.78	348	53.46	584	47.48	614	42.88	<0.0001	<0.0001
**Single mother**												
**Belgium**	4 191	15.63										
**Maghreb**			331	8.90	479	10.31	792	12.94	1181	16.52	<0.0001	0.10
**Sub-Saharan Africa**			515	38.90	672	40.02	434	38.34	206	31.69	0.04	<0.0001
**Turkey**			34	6.43	104	12.62	227	15.25	243	15.90	<0.0001	0.17
**LBW**												
**Belgium**	1447	5.4										
**Maghreb**			107	2.9	144	3.1	210	3.4	300	4.15	0.003	<0.0001
**Sub-Saharan Africa**			64	4.8	102	6.1	81	7.0	52	7.8	0.04	0.006
**Turkey**			21	4.0	35	4.25	82	5.55	79	5.1	0.42	0.69

There is a higher proportion of single mothers among Belgians (15,6%) compared to Maghrebi and Turkish mothers (8,2% and 6,9% respectively for non-naturalized mother) (Table 1). Among these two groups, the proportion of single-motherhood increases significantly with time since naturalisation (p<0,0001) and after 10 years of naturalization the rates are similar to the rate of Belgians (Table 2): 8,9% to 16,5% (Maghreb) and 6,4% to 15,9% (Turkey). The pattern is different for sub-Saharan African mothers. The proportion of single mothers (38,9% for non-naturalized mother) is significantly higher than that of Belgians (p<0.0001) and it slightly decreases with time since naturalisation (38,9 to 31,7% after 10 years of naturalization) (p = 0.04).

### The risk of low birth weight increases with time since naturalization

Table 3 shows the prevalence of LBW among Belgian and immigrant mothers (whether naturalized or not) and the risk of LBW according to immigrant nationality and time since having acquired the Belgian nationality. The results show that the risk of LBW among immigrants, compared to Belgians, varies according to the immigrant group. Concerning the time since naturalisation, we observe a similar pattern among all groups of immigrants: an increase of the risk of LBW after more than 1 year of naturalization. Similar results were observed for prematurity (not shown).

**Table 3:** Low birth weight (odds ratio and crude rates) according to naturalization length.

			*n = 75 175; cases = 3 507*		*n = 72 347; cases = 3 252*		*n = 61 872; cases = 2775*	
	Per 100 live births				*adjusted for maternal age*, *parity*, *maternal education and single motherhood*		*adjusted for maternal age*, *parity*, *maternal education*, single motherhood and income*	
	Total	%	OR (95% CI)	*p-value*	aOR (95% CI)	*p-value*	aOR (95% CI)	*p-value*
**Belgium**	26 918	5.38	1		1		1	
**Maghreb non-naturalized**	10637	3.74	0.68 (0.61–0.77)	<0.0001	0.63 (0.56–0.71)	<0.0001	0.57 (0.50–0.65)	<0.0001
naturalized <1 year	3692	2.9	0.53 (0.43–0.64)	<0.0001	0.54 (0.44–0.66)	<0.0001	0.43 (0.31–0.60)	<0.0001
naturalized 1<5 year	4643	3.1	0.56 (0.47–0.67)	<0.0001	0.56 (0.46–0.67)	<0.0001	0.51 (0.42–0.63)	<0.0001
naturalized 5<10 year	6107	3.44	0.63 (0.54–0.73)	<0.0001	0.62 (0.53–0.72)	<0.0001	0.54 (0.46–0.65)	<0.0001
naturalized ≥10 year	7225	4.15	0.76 (0.67–0.87)	<0.0001	0.76 (0.66–0.87)	<0.0001	0.71 (0.61–0.82)	<0.0001
**Sub-Saharan Africa non-naturalized**	4936	6.00	1.12 (0.99–1.28)	0.08	0.99 (0.86–1.04)	0.95	0.96 (0.82–1.12)	0.62
naturalized <1 year	1343	4.77	0.88 (0.68–1.14)	0.33	0.80 (0.61–1.05)	0.11	0.66 (0.44–0.99)	0.05
naturalized 1<5 year	1680	6.07	1.14 (0.92–1.40)	0.22	1.01 (0.81–1.26)	0.92	0.95 (0.75–1.21)	0.67
naturalized 5<10 year	1153	7.03	1.33 (1.05–1.68)	0.02	1.18 (0.92–1.51)	0.19	1.11 (0.86–1.43)	0.43
naturalized ≥10 year	666	7.81	1.49 (1.12–1.99)	0.01	1.20 (0.88–1.65)	0.25	1.01 (0.78–1.54)	0.60
**Turkey non-naturalized**	1812	4.91	0.91 (0.73–1.13)	0.40	0.93 (0.74–1.17)	0.54	0.80 (0.62–1.03)	0.08
naturalized <1 year	527	3.98	0.73 (0.47–1.13)	0.16	0.68 (0.42–1.11)	0.13	0.35 (0.13–0.95)	0.04
naturalized 1<5 year	823	4.25	0.78 (0.55–1.10)	0.16	0.80 (0.55–1.14)	0.22	0.75 (0.50–1.12)	0.16
naturalized 5<10 year	1477	5.55	1.03 (0.82–1.30)	0.77	1.05 (0.83–1.33)	0.69	1.09 (0.85–1.41)	0.50
naturalized ≥10 year	1536	5.14	0.95 (0.76–1.20)	0.69	0.96 (0.75–1.21)	0.71	0.92 (0.72–1.17)	0.51

*Without 2004

Maghrebi mothers (naturalized or not) have lower rates of LBW compared to Belgian mothers (p<0,0001): 5,4% of Belgian mothers compared to 3,7% of non-naturalized mothers from Maghreb. We observed that LBW varied according to time since naturalisation. It is lowest for women naturalized less than one year before giving birth (2,9%); then it increases significantly with the Belgian naturalization length up to 4,1% after more than 10 years of naturalization (p<0,0001) (Table 2). Yet, after more than 10 years of naturalization, LBW rates remain lower among immigrants compared to Belgians (p<0,0001). Among sub-Saharan African mothers, we observe slightly higher rates of LBW compared to Belgian mothers: 6% versus 5,4% for non-naturalized sub-Saharan Africa mother. Among Maghrebi mothers, the prevalence of LBW is lowest for mothers having recently acquired the Belgian nationality (4,8%) and then increases significantly with time since naturalisation (p = 0,04). After more than 10 years of naturalization LBW rates for women from Maghreb are significantly higher than for women of Belgian origin: 5,4% versus 7,8% (p = 0,01).

Compared to mothers of Belgian origin, Turkish mothers (naturalized or not) have slightly lower rates of LBW: 5,4% versus 4.9% for non-naturalized Turkish mothers. As with the other migrant groups, we observed that LBW declined just after the acquisition of the Belgian nationality (4%) and then increased (to 5,1% after more than 10 years of naturalization). This increase is smaller than that observed among mothers from Maghreb or sub-Saharan Africa (p-trend not significant).

After adjustment with maternal characteristics (parity and maternal age), the results did not change significantly, and odds ratios remained similar to the unadjusted ratios (not shown). After adjustment with maternal characteristics and SES indicators, the pattern of LBW according to time since naturalisation is maintained and even accentuated for all migrant groups: LBW rates are lowest for mothers who have recently acquired the Belgian nationality and increase thereafter. When including SES in the adjustment variables, mothers originally from Maghreb have an even lower risk of having a baby with LBW compared to mothers of Belgian origin; and in sub-Saharan African naturalized mothers the excess risk disappears. For Turkish mothers, we observed a significantly lower risk of LBW among women who acquired the Belgian nationality less than 1 year before giving birth compared with mothers of Belgian origin (p = 0,04).

## Discussion

To our knowledge, this is the first study using a Belgian database linking data on birth and death registers, migration trajectories and socio-economic characteristics of parents, which allowed analyzing the influence of both factors on the health of children at birth. The main result of this study is that in all migrant groups LBW increases with the time since naturalization despite an improvement of maternal SES. More specifically, LBW follows a U-curve: mothers who acquired the Belgian nationality less than one year before giving birth have lower rates of LBW than those who did not acquire the Belgian nationality. Then, the LBW rates increase significantly with the time since naturalization, in all immigrant groups. Interestingly, after 10 years of naturalization mothers originally from Maghreb still have lower rates of LBW compared to mothers of Belgian origin.

### A curvilinear association between naturalization length and LBW

The apparent curvilinear pattern observed in this study is in line with several studies showing that a long duration of residence is negatively associated with health. Study carried out in Canada found that recent immigrants were at lower risk of preterm birth compared to the Canadian-born population, but immigrants became at higher risk after 10 years of stay in Canada [9]. Similar results have been shown for Hispanic immigrants in the US, where the rates of LBW appeared to decline over the first few years of residence and increase thereafter [20]. Data from Sweden also found a curvilinear association between birthweight and most HDI (Human Development Index) categories by maternal duration of residence [19]. This curvilinear pattern has also been observed for other health outcomes, with improvements in health among immigrants soon after arrival followed by a steady deterioration after a few years in the native country [28].

The “epidemiological paradox” is a common explanation for this observation. Despite their low SES, immigrants are in better health than native women [23,29,30]. In our study, another hypothesis to explain this improvement in health when giving birth less than one year after acquiring the Belgian nationality could be linked to the improvement of the social and psychological situation at the moment of naturalization, in terms of living situation, employment, and family situation is probably. The access to host-country citizenship is often seen as an important element in the integration process and is more likely to be granted to people who have higher education, legal status and better language skills.

### Acculturation paradox

One explanation that has been put forward by other authors for a deterioration of health with time spent in the host country is the “acculturation paradox ». This can be explained by the combination of two factors. On one hand, immigrants tend to adopt risky health-related behaviours from the host population, such as smoking, alcohol consumption, and consumption of energy dense food [21]. On the other hand, the loss of social and cultural protective factors erodes their health over time [21,31]. In our study we also observe an increase of single parenthood with naturalization length among Maghrebi and Turkish mothers and the rates are similar to mothers of Belgian origin after 10 years since naturalization. The observed increase of single parenthood could be part of the acculturation process. Moreover, we know that maternity out of wedlock is rare among certain communities and can be a source of stigma [32].

Another hypothesis to explain a deterioration of health with time spent in the host country is the exposure to an accumulation of social stressors such as stigmatisation, discrimination, poor housing conditions, unemployment, or poor working conditions [33–36]. In general, citizenship acquisition is associated with better labour market outcomes for non-Western immigrants [37] But the existence of structural racism means that immigrants continue to face many barriers in accessing work and particularly accessing stable jobs that match their qualifications. The rates of unemployment are higher among immigrants than among native populations [38–40] and they are exposed more frequently to unstable employment and poor working conditions, which might particularly affect the health of pregnant women. Migrants are likelier to suffer from poor housing conditions and from discrimination that both generate global stress that can have important consequences on health. Social stressors experienced by immigrants as racism [41] and islamophobia [42] have been shown to be negatively associated to health and socioecological determinants of health.

### An increase of LBW with time since naturalization, despite an improvement of SES

In our study, we observe an improvement of maternal SES with time since naturalization among all migrant groups; yet, rather than being linked to a decrease in LBW, this improvement of social and economic conditions is associated with an increase of LBW. Other studies have shown that improvements of SES are not always accompanied by better health [8,43,44]. For example, a study in Canada observed a higher proportion of preterm birth and morbidity during pregnancy among long-term immigrants despite their higher incomes and better support during pregnancy compared to recent immigrants [8].

This result could be explained by the fact that the impact of socioeconomic indicators we frequently study on health outcomes is less important among immigrants than among native populations. Some studies have observed a weak or flat social gradient in health among immigrants [13,24,29,43,45–48]. Unlike the native population, where an increase of SES is associated with better health, this gradient is not present among immigrants. For example a study has shown that the protection against LBW varies considerably by maternal education across ethnic groups [45]. In Brussels, there is a protective effect of migration in relation to perinatal mortality and LBW among women with low SES but not among women with high SES [18]. One of the plausible explanations is the lacking link between better SES and better living conditions for migrants. In other words, at the same level of qualification, immigrants are less likely to access the same quality of employment or housing as the native population. We also suppose that with the same education level and income, the mothers do not have the social status in their own country than in the host country.

### Mothers from Maghreb maintain their protection against LBW even after more than 10 years of naturalization

Another important result of our study is that, as opposed to mothers from Turkey or sub-Saharan Africa, after 10 years of naturalization, mothers from Maghreb still have lower rates of LBW compared to Belgian mothers. This is consistent with previous studies where some migrant groups but not others were protected for certain outcomes. In Belgium, some migrant groups (Maghreb, Turkey) are protected for certain outcomes (prematurity and LBW) but are at risk for others (perinatal mortality) [15,18]. Indeed, some risk factors for adverse perinatal outcomes appear to be specific to certain migrant groups. LBW among Sub-Saharan African women is associated with a higher incidence of hypertension and genito-urinary infections whereas perinatal mortality among Maghrebi and Turkish women is associated with higher prevalence of foetal or newborn deaths caused by congenital anomalies [14,49,50].

Women from Maghreb are protected against LBW, but the pattern is different for perinatal mortality. Previous studies have shown that the perinatal mortality rates decrease significantly for mothers having acquired the Belgian nationality [14]. Because our linked database concerned live births only it was not possible to study perinatal mortality according to naturalization length. A study in Italy also who found that perinatal outcomes improved over time in some immigrant women [51]. It seems that, depending the perinatal outcome studied, we have an improvement or a decrease of perinatal health according to time since naturalization.

### Limitations

This study has some limitations. A limitation of our study is that we lack the duration of residence, meaning that we don’t have the duration of residence before naturalization nor the duration of residence for women who have not acquired the Belgian nationality. The time since naturalization might be considered a proxy for the duration of residence for the immigrants that have acquired the Belgian nationality, but it has some important limitations since the duration of residence is not the only factor considered in naturalization applications. For example, some women may have resided in Belgium for a long time but not be granted naturalization for other reasons or may not have applied for it. As explained in the methodology, it is difficult to estimate the length of residence before the acquisition of the Belgian nationality. The acquisition of the nationality might reflect better migrant integration, ie. better access to the labour market, to social and health services, higher education, better housing conditions and political participation [52]. A Belgian study shows that people from non-Western countries, especially women, are more likely to be employed if they have acquired the Belgian nationality and this observation is not affected by the length of residence. The authors point out, however, that it is difficult to isolate acquisition of nationality and length of residence since they are strongly correlated [37]. Both length of residence and time since naturalisation are linked to immigrant integration.

We also lack information on the use of antenatal care. We know that adverse perinatal outcomes among immigrants are associated with late or inadequate prenatal and obstetric care, possibly due to the difficulty of accessing services [53,54]. In Brussels, a study is ongoing which explores migrant and non-migrant women’s access, use and experience of perinatal healthcare services, which might shed more light on this matter. We do not have any information on preconception health care, which also influences perinatal health outcomes such as LBW [55]. Access to preconception health care may improve over time to and may partly explain the decline in LBW with increasing time since naturalization.

Another limitation concerns the lack of data on some important predictors of LBW such as tobacco use, hypertension, alcohol consumption, and maternal BMI. For example a Belgian study has observed the association between height and the risk of preterm birth was modified by maternal nationality [56].

## Conclusion

This study demonstrates the importance of considering the time since naturalization as a predictor of perinatal health outcomes among immigrants. Among all the groups of immigrants studied, we observe a significant U-shape increase of LBW despite an improvement of maternal SES. Maghrebi mothers maintain their protection against LBW even after more than 10 years of naturalization. This study illustrates also the importance of linking administrative databases for the analysis of social determinants of perinatal health. Additional studies need to be carried out in order to gain a better understanding of the association between migration trajectories, SES and birth outcomes and the mechanisms that lead to perinatal health inequalities.
